# WEDM of Copper for the Fabrication of Large Surface-Area Micro-Channels: A Prerequisite for the High Heat-Transfer Rate

**DOI:** 10.3390/mi11020173

**Published:** 2020-02-07

**Authors:** Naveed Ahmed, Mohammad Pervez Mughal, Waqar Shoaib, Syed Farhan Raza, Abdulrhman M. Alahmari

**Affiliations:** 1 Department of Industrial and Manufacturing Engineering, University of Engineering and Technology, Lahore 54890, Pakistan; mp_mughal@hotmail.com (M.P.M.); waqarshoaib888@hotmail.com (W.S.); sf.raza.rezvi@gmalil.com (S.F.R.); 2 Industrial Engineering Department, College of Engineering and Architecture, Al-Yamamah University, Riyadh 11512, Saudi Arabia; 3 Raytheon Chair for Systems Engineering, Advanced Manufacturing Institute, King Saud University, Riyadh 11421, Saudi Arabia; alahmari@ksu.edu.sa

**Keywords:** wire electrical discharge machining (WEDM), discharge energy, micro-channels, deflection, copper, fin-thickness, fin-radius, fin-height, surface area, heat transfer

## Abstract

To get the maximum heat transfer in real applications, the surface area of the micro-features (micro-channels) needs to be large as possible. It can be achieved by producing a maximum number of micro-channels per unit area. Since each successive pair of the micro-channels contain an inter-channels fin, therefore the inter-channels fin thickness (IFT) plays a pivotal role in determining the number of micro-channels to be produced in the given area. During machining, the fabrication of deep micro-channels is a challenge. Wire-cut electrical discharge machining (EDM) could be a viable alternative to fabricate deep micro-channels with thin inter-channels fins (higher aspect ratio) resulting in larger surface area. In this research, minimum IFT and the corresponding machining conditions have been sought for producing micro-channels in copper. The other attributes associated with the micro-channels have also been deeply investigated including the inter-channels fin height (IFH), inter-channels fin radius (IFR) and the micro-channels width (MCW). The results reveal that the inter-channels fin is the most critical feature to control during the wire electrical discharge machining (WEDM) of copper. Four types of fin shapes have been experienced, including the fins: broken at the top end, deflected at the top end, curled bend at the top, and straight with no/negligible deflection.

## 1. Introduction

Rapid expansion is being noticed in the area of miniaturized manufacturing. Micro-molds, micro-heat exchangers, micro-gears, and micro-reactors are common examples of manufacturing applications in the area of miniaturized engineering. Such applications usually require transferring the heat from one side of the component to the opposite end by the flow of different fluids. Heat transfer is carried out through various types of micro-fluidic structures/features, amongst which the micro-channel is the most widely used feature [1,2]. Various types of materials can be found to be used for the fabrication of micro-channels including metals, alloys, and non-metals.

Among the category of metallic micro-channels, copper is one of the widely used material because of its good thermal characteristics which play a significant role during heat transfer [3,4,5]. Qin et al. [6] have fabricated an evaporator-collector, used in the pump of an aqueous heater, made up of copper fins having a thickness of 350 µm. Kussul et al. [7] fabricated a micro-channels recuperator made up of copper and used in the Ericsson engine. They improved the engine efficiency based on the low-temperature difference (5 °C) achieved after designing copper micro-channels which was otherwise not possible.

Larger surface-area density is one of the primary concerns for efficient heat transfer applications. It indicates that the higher the amount of surface area of the micro-channels, the larger the amount of heat that can be transferred. Compact heat exchangers (CHEs) are examples of such applications [8]. Aspect ratio, outside surface area, and surface-to-volume ratio are the micro-channels’ characteristics that affect the fluid flow rate [9]. Larger surface areas offer high reaction rates per unit volume of the chemical reactors [10]. Porous structures are considered the other alternatives for achieving high surface area [11]. Another way of enhancing the surface area is the use of different shapes of the micro-channels, e.g., plain, wavy, and interrupted fin [12]. Likewise, the use of V-shaped ribs arranged in a periodic order can augment the surface area [13] to improve the performance of micro-channels [14]. The saw-toothed profile is another alternate for high surface area [15].

Micro-milling, lithography, embossing [16], ultra-precision machining, multi-cutter milling [17] and [5], laser beam machining (LBM), electrochemical machining (ECM) [18], photochemical machining (PCM) [19], hybrid machining [1], and some others are the reported methods to produce micro-channels on variety of substrate materials. PCM, LBM, and ECM are very well-known processes to produce very thin micro-channels (even down to 100 µm) but these processes are not very suitable to achieve high aspect-ratio micro-channels [20]. Thus, a compromise on large surface-area requirements is the prominent limitation of the above processes. Another shortcoming of the LBM is the re-deposition of melt spatter inside or around the previously machined micro-channel, that ultimately affect the precision of the micro-channels [21]. Deformation in the micro-channel’s fins is the potential limitation of ultra-precision machining [22]. Punch and die stamping can also be used to produce the micro-channels but not the deeper ones [23]. Hence, it can be inferred that most of the processes are suitable for low aspect-ratio machining of micro-channels or the processes induce certain defects in the machined features.

The wire-cut variant of electrical discharge machining (EDM) is very widely used process in manufacturing industry. To cut simple-to-complicated profiles in variety of conducting materials is a core competency of wire electrical discharge machining (WEDM) [24]. The WEDM is well-known for cutting complicated contours on numerous types of metallic materials, however, the profile inaccuracies and the error at the sharp corners are the matters of concerns [25]. The traditional wire-cut EDM could not be widely seen to be used for the cutting of micro-channels, in particular. The micro-electric discharge milling can, however, be used for machining the micro-channels [26]. The tooth thickness of a miniaturized gear can be considered as a similar feature as the inter-channels fin thickness in micro-channels. WEDM can be easily used to cut the macro-sized gears but the cutting of miniaturized gear is relatively difficult [27]. Moreover, producing a single micro-feature through WEDM is simple but the cutting of repeated micro-features (micro-arrays) is reasonably difficult [28]. Miller et al. [29] produced thin structures through WEDM and deformation in the thin section has been reported as the drawback. 

From the literature review, it can be witnessed that several techniques are used to fabricate micro-channels. Amongst these techniques, the use of WEDM is not found to be very common. Larger surface areas are reported as the desired feature to achieve higher rates of heat transfer. The larger surface area can be achieved by: (1) high aspect-ratio micro-channels, (2) greater height of inter-channels fins, (3) reduced fin thickness, and (4) increased number of fins per unit area (fin density). The main advantage of WEDM is that it may produce substantially deeper channels (high aspect ratio) since the cutting tool is a continuously moving wire electrode that has absolutely no limitation to penetrate the substrate which otherwise is a limitation in the case of multi-cutter milling, ECM, and precision machining etc. High aspect ratio ensures the large surface area. 

Therefore, in this research, the fabrication of micro-channels on the copper substrate has been realized through WEDM. The focus is kept on achieving the micro-channels having large surface area. If the thickness of the inter-channels fin is smaller, a large number of micro-channels per unit area can be machined. Thus, the performance of WEDM has been evaluated in terms of four response characteristics associated with the micro-channels including the inter-channel fin thickness (IFT), inter-channels fin height (IFH), inter-channels fin radius (IFR), and micro-channel width (MCW). These response measures are evaluated against the five parameters of WEDM. The minimum values of IFT, IFR, and MCW, and the maximum value of IFH will eventually lead to having the maximum number of micro-channels per unit area of the substrate. The process parameters affecting each of the four responses are ranked based on the results of signal-to-noise ratio analysis. In this way, the process control can be realized to achieve the desired micro-channel feature.

## 2. Materials and Methods 

**Figure 1:**
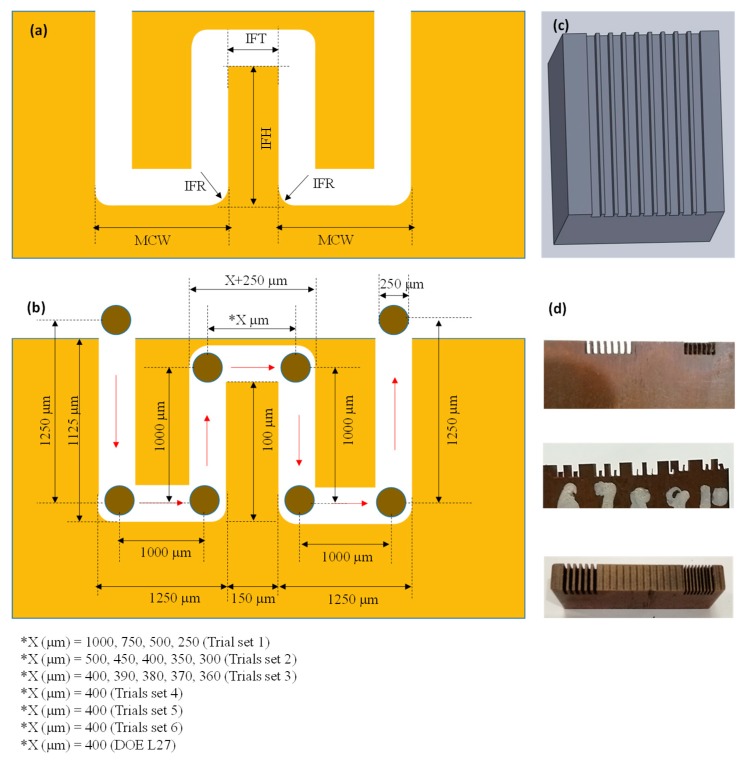
Micro-channels fabricated through wire electrical discharge machining (WEDM): (**a**) schematic of responses, (**b**) schematic of micro-channel features with dimensions, (**c**) 3D computer-aided design; CAD model of the designed micro-channels, and (**d**) actual machined samples.

The use of copper is very common is several applications wherein heat transfer is required. Such applications include heat exchangers (macro and micro), chemical reactors, heat plates and heat sinks, and many others. Flow dynamics and other associated characteristics of the fluid flow depend on the geometry and precision of the micro-features such as the micro-channels. There are many manufacturing processes available that are being used to generate micro-channels such as chemical etching, electrochemical machining, laser machining, and micro-machining. Thus, in this research work, wire electric discharge machining is used to fabricate micro-channels in the copper substrate. A copper sheet of 5 mm thickness is firstly cut into pieces of 50 mm length and 25 mm width. Some selected properties (thermal and physical) of the copper substrate are presented in Table 1. Before performing the machining, the top, bottom and side surfaces were ground to achieve a flat surface at each side. Serious attention was paid to clamp the workpiece on the machine table in such a way that the frontal work surface is perfectly parallel to the wire electrode and the workpiece top surface is perpendicular to the axis of the wire electrode. The tool electrode used in this study is the brass wire (0.25 mm diameter) continuously traversing along the vertical axis, i.e., fresh wire is continuously fed to produce the cut. During the electric discharge cutting, the resistivity of the medium was continuously checked by resistivity meter and kept constant during all the experiments. From the list of WEDM parameters, five commonly used parameters are taken as variables including pulse on-time (T-on), pulse off-time (T-off), servo voltage (SV), wire tension (WT), and wire feed rate (WF). Each variable is varied within three levels as shown in Table 2. The selection of these variables and their levels is carried out after evaluating their effects on micro-channel geometry through extensive trials sets (29 runs). The detailed results of the trials are discussed in the next section. The performance of the WEDM is evaluated in terms of four response characteristics associated with micro-channels. It includes the micro-channel width (MCW), inter-channels fin height (IFH), inter-channels fin thickness (IFT), and inter-channels fin radius (IFR). The responses are schematically shown in Figure 1a whereas the 3D computer-aided design (CAD) model of the micro-channels is shown in Figure 1c. After performing trial runs and recording the observations, mature experimentation was carried out under the Taguchi L27 design of experiments. Under L27 design of experiments (DOE), a square cross-section is taken as the designed geometry of the micro-channels. The measurements of each response characteristic were performed through coordinate measuring machine having a measurement resolution of 1 µm. For each experimental run, the measurements associated with each response were taken and repeated three times. The average values of measurements are reported. For example, the IFT was measured at three different points alongside the height of inter-channel fin and the average value is calculated for analysis purposes. In a similar way, the micro-channel width, inter-channel fin height, and inter-channel fin radii were recorded. The effects of each of the five machining variables on every individual response attribute were analyzed through main effect plots. Interaction effects plots are further developed to find out the possible contribution of two mutually-interacting variables on the micro-channel features. Analysis of variance (ANOVA) performed for every individual response helped to recognize the importance of process variables with respect to the WEDM performance measures. Correlation analysis is performed in detail to access the relationship of each of the five parameters with each of the set performance measures (4 measures; IFT, IFH, IFR, and MCW). Signal-to-noise ratio analysis is also conducted to seek and to prioritize the machining factors based on their ranking so that the most important factors affecting the micro-channel features can be identified.

## 3. Results and Discussion

### 3.1. Trial Experimentation (Phase-1)

**Figure 2:**
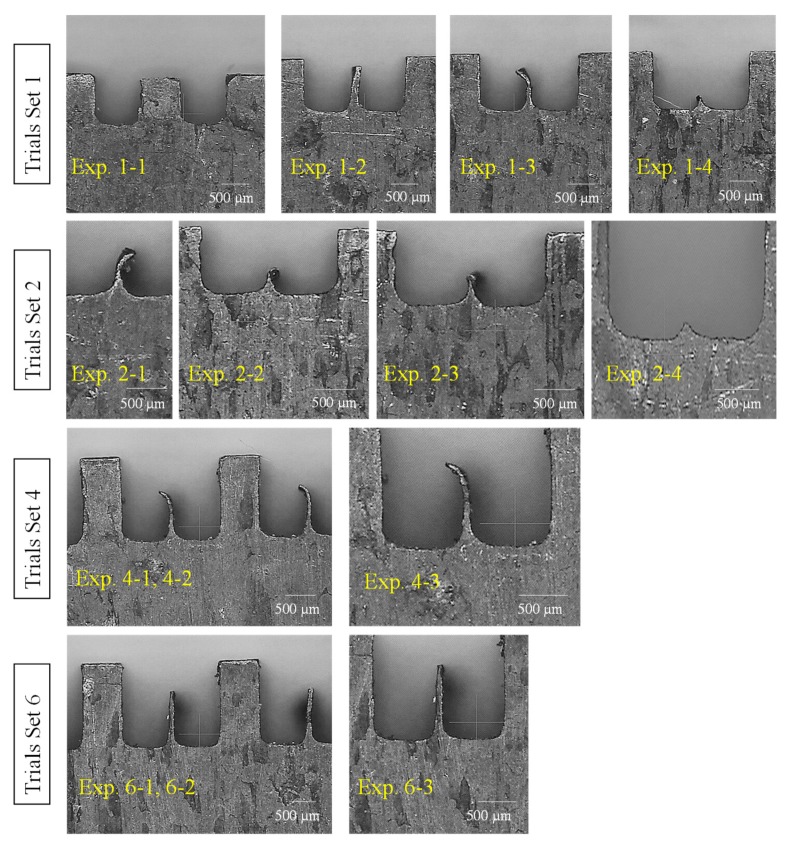
Experimental results of different trial sets for determining the minimum inter-channel fin thickness.

Since in the literature, WEDM has not been commonly found as a process to fabricate micro-channels in copper, therefore firstly trial runs were performed. Having no guidelines regarding the minimum possible channel size achievable during WEDM, six trial sets were designed. Each trial set contains multiple experimental runs in which sizes of micro-channels and inter-channels fin thicknesses were systematically varied. The detail of these trial sets is presented in Table 3. For example, in the first trial set four experiments were performed in which fin thickness was varied with an increment of 250 µm, i.e., the values of designed IFTs were 1000, 750, 500, and 250 µm. The machining parameters were kept constant for this trial set. Measurements of IFTs were recorded and the shape of the inter-channels fins observed. For the channels with IFT 1000, 750, and 500 µm, the actual fins retained their shape (rectangular cross-section), whereas the channels with IFT = 250 µm diminished under the action of machining phenomenon. From these observations, the second trial set was planned in which the IFT was kept between 500–300 µm with a progressive decrease of 50 µm. In this way, five experimental runs were performed having IFT values of 500, 450, 400, 350, and 300 µm. The channels with IFT ≥400 µm retained their shape, however, the micro-channels with IFT ≤350 µm lost their geometry (no evidence of fin existence) as can also be witnessed in Figure 2 (fourth image in 2nd row; experiment 2-4). Therefore, the third trial set having five experimental runs was planned and executed in which the inter-channel fins were designed with thicknesses reducing from 400 µm to 360 µm with a progressive difference of just 10 µm. Again the single large-channels (sum of two channels) were produced with no evidence of inter-channel fin in case of IFTs ranging from 360 to 390 µm. The inter-channel fin persevered its existence only in the case of IFT = 400 µm. In order to make sure about this result, a fifth trial set was performed under the same machining conditions and the designed IFT value of 400 µm was repeated. No measurements were performed but the microscopic-based visual observations were noted. It was noticed that in all these 5 runs the inter-channels fins were present but each fin has a bend at the upper tip point as can be seen in Figure 2. Thus, the set of trials was repeated by varying the WEDM parameters (higher values of machining parameters than the previously used values) but keeping the designed IFT constant at 400 µm. In Table 3, this set is named as trials set 5. Each experimental run of this trial remains unsuccessful in terms of generating micro-channels and inter-channel fins with desired geometry. So far, it was confirmed that micro-channel size should be designed in such a way that inter-channels fin should have a minimum thickness of 400 µm. Hence, another trial set 6 was attempted in which the parametric values of WEDM variables were again changed (values less than the values in the cases of trial sets 1-4). Finally, the micro-channels having straight inter-channel fins were successfully achieved with a slight deflection. Hence, based on the findings of such extensive trial experimentation the mature experiments were designed through the Taguchi L27 to evaluate the influence of WEDM variables on the remaining attributes associated with the micro-channels, i.e., IFH, IFR, and MCW.

### 3.2. Mature Experimentation under Taguchi L27 (Phase-2)

**Figure 3:**
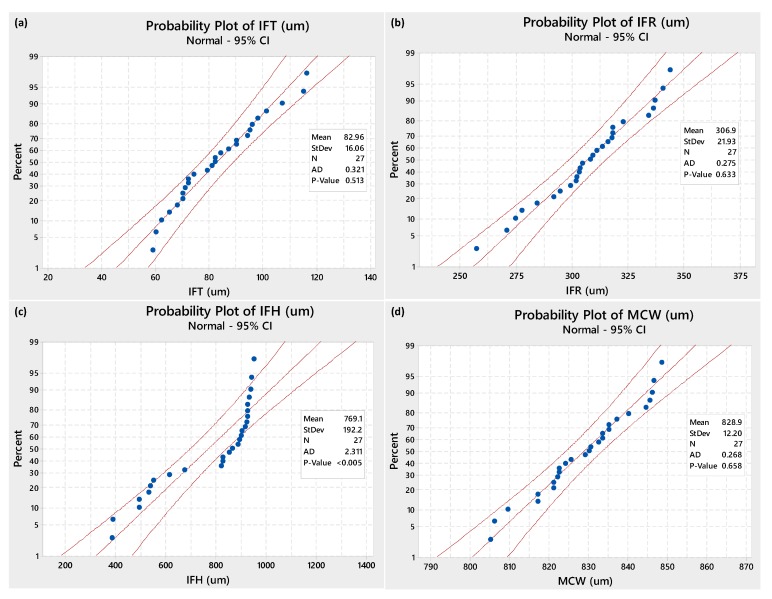
Probability plots of data points associated with: (**a**) inter-channels fin thickness (IFT), (**b**) inter-channels fin radius (IFR), (**c**) inter-channels fin height (IFH), and (**d**) micro-channels width (MCW).

**Figure 4:**
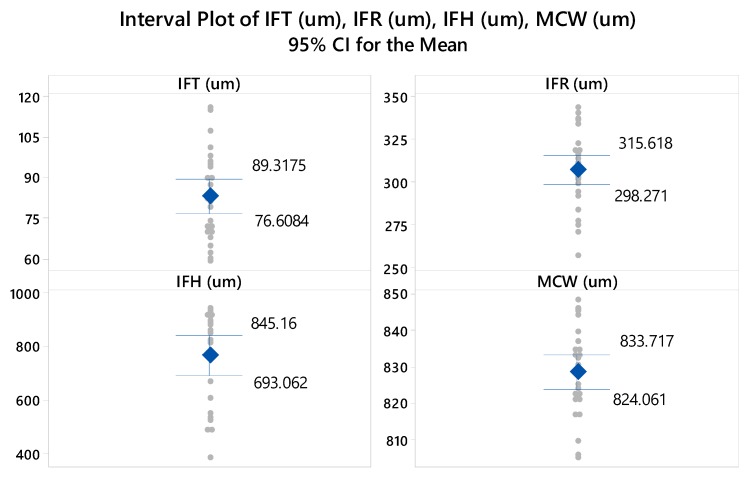
Interval plots for means of responses drawn at 95% confidence interval (CI).

After the first phase of trial sets (29 experimental runs), the second phase of experimentation was executed by following Taguchi L27 experimental design. The experimental results in terms of micro-channels’ attributes (IFT, IFR, IFH, and MCW) are presented in Table 4. By scanning the results table, a significant variation can be noticed in the columns of responses. For example, among the 27 experimental runs, the inter-channels fin thickness (IFT) acquires a minimum value of 59 µm and a maximum one of 116 µm. Likewise, the inter-channels fin radius (IFR) varies from 257 µm to 343.5 µm, inter-channels fin height (IFH) suffers from huge variation having a minimum of 383 µm and a maximum of 949 µm height, and the micro-channel width ranges within 805–845.5 µm. To further facilitate, descriptive statistics containing the important details of data are presented in Table 5. Moreover, the probability plots (as presented in Figure 3) for the data associated with each of the set responses are developed using a 95% confidence interval. The experimental results data points corresponding to three out of four responses naming IFT, IFR, and MCW are closel to the good-fit line. The P-values of the said three responses are >0.05 (threshold value) indicating that the data points referring to IFT, IFR, and MCW follow a normal distribution. For the inter-channels height (IFH) the data spread doesn’t follow the normal distribution as indicated by the plot shown in Figure 3 as well as the p-value which is less than the threshold value. The mean values of each response characteristic, as presented in Table 5, fall inside the 95% confidence intervals of the means as can be seen in Figure 4.

### 3.3. Parametric Effects and Signal-to-Noise Ratio Analysis

#### 3.3.1. Analysis of Interchannels Fin Thickness (IFT)

**Figure 5:**
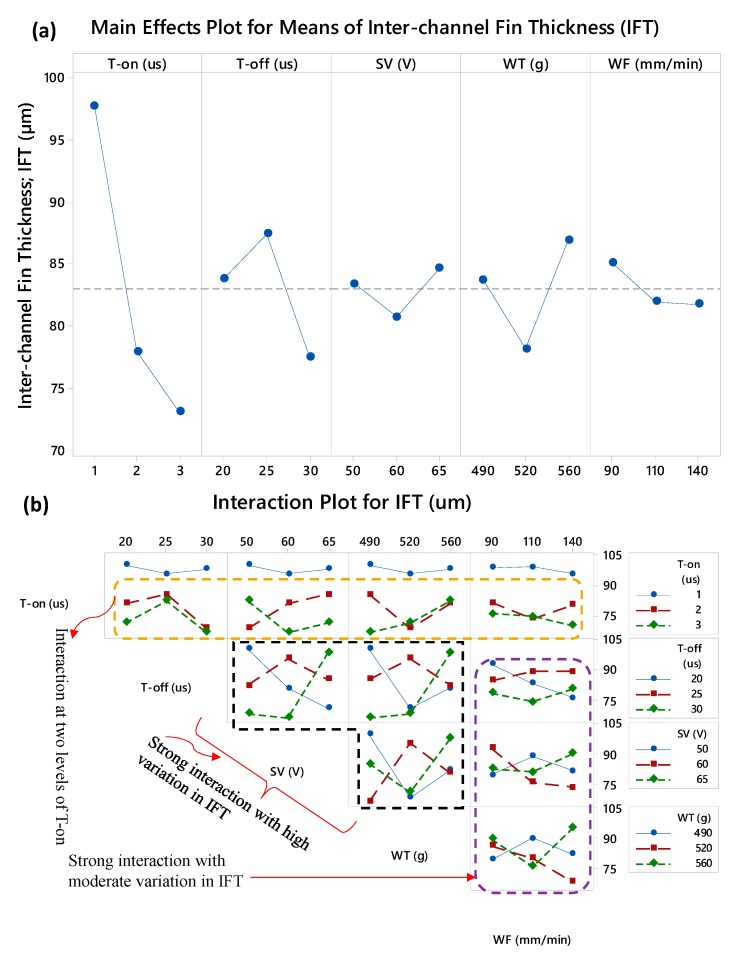
Parametric effects on inter-channel fin thickness (IFT): (**a**) main effects plot and (**b**) interaction plot.

The dependence of micro-channels’ attributes over the machining parameters has been examined with the help of main effects plots and the interaction plots. Figure 5 shows the corresponding plots developed for the inter-channels fin thickness (IFT). The effect of on-time of the discharge pulses on IFT is exceptionally sound if compared with the effects of other parameters. As the pulse on-time increases the fin thickness significantly reduces. The mean value of IFT corresponding to the experiments performed with 1 µs pulse on-time is 97 µm whereas the on-time of 3 µs results a mean value of IFT equals to 73 µm. To generate the inter-channels fin, the wire electrode first travels along the upward direction by eroding the material from the left side of the fin as schematically shown in Figure 1b. As the wire electrode completes its travel along the one side (left side) of the fin and returned back along the downward direction on the other side (right side) the erosion of material also occurred. In this way, the inter-channel fin is developed. The sum of the material removal from both sides of the fin decides the resulting fin thickness. In the case of 3 µs on-time, the time interval for the pulse to interact with the work surface increases and more material is removed. High material removal from both the side-walls of the fin allows the process to generate a thinner fin. It has been reported in the literature that while machining of thin sections through WEDM the trends of the process variables effects do not remain obvious as compared with the trends in the case of WEDM of thick sections [25]. The teeth on the miniaturized gears can be considered as similar features as the inter-channels fins. It has been reported that the low discharge energy produced due to the lower value of T-on is the most appropriate to preserve the geometry (tooth thickness) of the gear teeth. Along with the other parametric combinations, the pulse on-time of 1 µs has been recommended to obtain high-quality brass gears manufactured by WEDM [30]. The influence of discharge pulse’s off-time (T-off) on IFT is such that the IFT first increases and then tends to reduce with the rise in T-off. A mid-level of T-off seems to be favorable to achieve high values of IFT. The effects of servo voltage (SV) and wire feed (WF) are relatively small and no significant variation in IFT is observed. The tension induced in the wire electrode, however, imparts a certain noticeable effect on IFT. The higher level of wire tension (560 g) is more promising to obtain thicker fins.

In order to get a prioritized pattern of machining parameters with respect to IFT, signal-to-noise ratio (S/N) analysis has been performed and the results are presented in Table 6. Since the fin thickness plays a significant role in applications and the more is the fin thickness the higher will be the strength per unit area. Therefore, larger the better criterion was set for S/N ratio analysis. The high value of delta in the S/N ratio supports prioritizing the rank of the contributing factor. Thus, based on the delta values calculated against each of the five machining parameters the ranking has been assigned. From Table 6, it can be inferred that the more attention should be paid towards the settings of T-on, T-off and WT, since these three parameters are ranked as 1, 2 and 3, respectively. However, the WF and SV received a 4th and 5th rank, respectively leading to infer that the least attention is required for these two variables if the objective is to have the inter-fins with larger values.

On the other end, the interactions of different machining parameters have a considerable role in the development of inter-channels fins as can be seen in Figure 5b. The non-parallel lines intersecting each other reveals the existence of interaction effect whereas the parallel or non-intersecting lines show no interaction effect. Pulse on-time at its first level (T-on = 1 µs) does not interact with any of the remaining four parameters since the line corresponding to 1 µs on-time does not intersect with the other trend lines as can be witnessed at the top row of Figure 5b. All the levels of T-off, SV, and WT have strong interaction with each other and, therefore, the patterns of IFT values are monotonic. The interaction of wire feed rate with other machining factors is found to be strong but the variation in IFT can be said as the moderate since the IFT varies within 72–92 µm. Ali and Mohammad [31] fabricated a tool for embossing operation and a micro-gear on the copper substrate. The micro-tool and the gear comprised of the successive fins or blades. The thickness of the blades is reported as of 500 µm obtained after the WEDM of copper.

#### 3.3.2. Analysis of Interchannels Fin Height (IFH)

The inter-channels fin height is the main attribute deciding the aspect ratio of large surface area micro-channels. Electrochemical machining is found to be promising method to produce micro-channels in copper foil with high geometrical accuracies [32]. However, high aspect-ratio micro-channels are challenging. The tool electrode needs to be fed inside the channel for chemical dissolution. Due to the penetration of the tool inside the channels the removal of freshly produced debris becomes difficult thus the limiting production of deeper micro-channels. In the case of WEDM the tool is a wire electrode, and due to the penetration of the wire along the cut and continuous movement of the wire the problem of debris ejection is minimized. The moving wire also takes some debris along the direction of its travel. Thus, the wire can make deeper cuts and as a result higher aspect ratio can be achieved endorsing the advantages of WEDM in the context of micro-channels fabrication. Zhou et al. [33] fabricated the micro-channels on copper through laser beam micro-milling and the depth of micro-channels is found to be limited due to the debris expulsion difficulty. 

**Figure 6:**
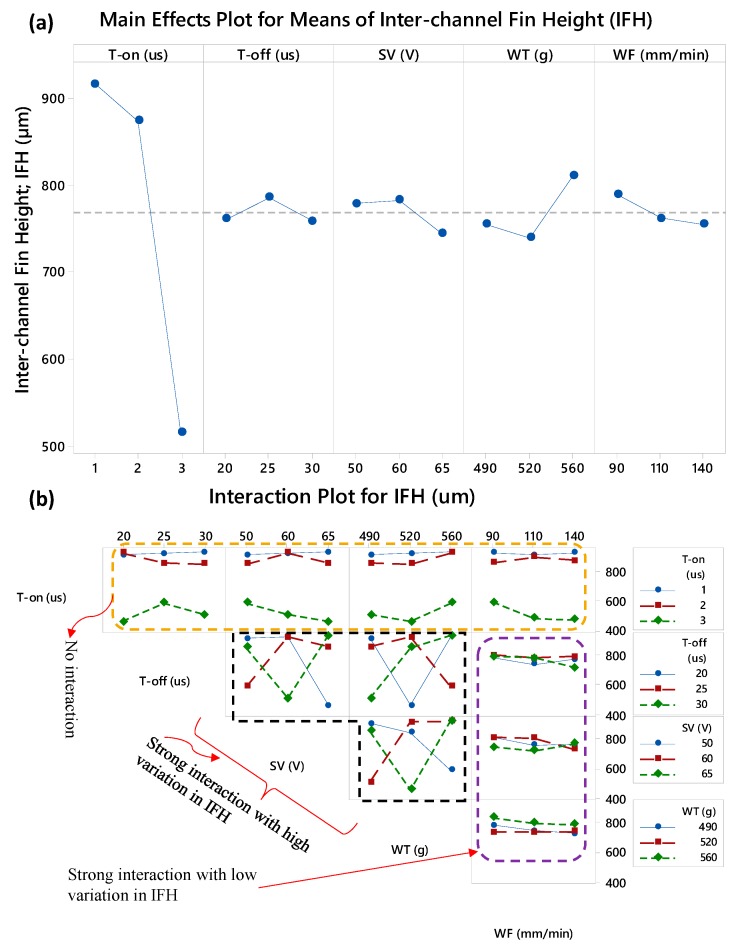
Parametric effects on inter-channel fin thickness (IFH): (**a**) main effects plot and (**b**) interaction plot.

The WEDM parametric effects on IFH in terms of main effects and interaction effects plots are presented in Figure 6. From the main effects plot, it can be witnessed that the IFH is mainly governed by the suitable value of T-on. The remaining factors (T-off, SV, WT, and WF), with respect to their individual performance, have no significant contribution in deciding the height of the machined fins since the slopes/variations caused by these variables are very minimal. The effect of WT is comparatively considerable since the mean value of IFH is high at 560 g of wire tension. The same observations are captured during S/N ratio analysis belongs to IFH in which the larger the better criterion was set to rank the machining parameters. From Table 7, it can be noticed that the delta value of T-on time is exceptionally high (5.11) as compared with the delta values of the remaining four factors. T-off, SV, and WF all have the delta values around 0.6. Thus, in the case of IFH, only the two machining factors can be considered as the most important, i.e., the pulse on-time and the wire tension which are ranked at 1st and 2nd position. However, the machining factors noticeably involved in terms of interaction effects. Three kinds of patterns with respect to interaction effects are observed in the case of IFH, i.e., no interaction, strong interaction with high variation in IFH, and strong interaction with low variations in IFH. The top row of Figure 6b shows that the trend lines of T-on are not intersecting with the trend lines of the remaining four parameters indicating that the pulse on-time has no interaction with the other machining parameters. On the other end, the T-off, SV) and WT strongly interact with each other and impart a significant change in the height of the produced inter-channels fins (IFH). The trend lines against the wire feed rate (WF) intersects among each other meaning that interaction does exit but the corresponding variation in IFH is on the lower range. Similar results are observed in the case of IFT.

**Figure 7:**
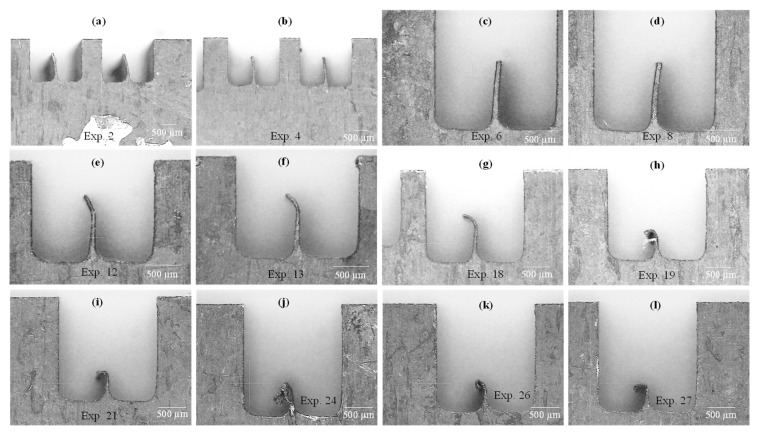
Micro-channels produced under different experimental runs of Taguchi L27.

The actual micro-channels along with inter-channels fins machined through WEDM are selectively presented in Figure 7. The machining results, especially in terms of IFH, are of three configurations: (1) the inter-channels fins are approximately straight with good IFH values (Figure 7a–d), (2) the inter-channels fins are of appreciable height but slightly deflected at the tip region of the fins (Figure 7e–g), and (3) low heightened inter-channels fins with curly tips (Figure 7h–i). Liao et al. [28] have stated that machining of single micro-feature is easier to produce with WEDM but the features arranged in a periodic order (micro-arrays) are difficult to produce via WEDM process. The low-speed wire-cut process has been recommended for producing micro-features of size ranging below 100 µm thickness. Miller et al. [29] also reported that WEDM develops a noticeable bend upon machining of thin cross-sections. They cut pure titanium and achieved a lowest thickness amounting to be 61 µm by the use of T-on = 2 µs and thickness of 165 µm using T-on = 18 µs. By evaluating the experimental results (shown in Table 4), the experimental runs 1 through 9 are performed with a mix of machining parameters but with pulse on-time having a constant value of 1 µs. Likewise, the experiments 10 through 18 have 2 µs T-on and the experimental runs 19 through 27 are executed under 3 µs T-on. Upon relating the images (shown in Figure 7) with the DOE and results in Table 4, as the intensity of the electric discharges increases through increasing the pulse on-time, the configuration of the inter-channels fins considerably changes. When the T-on is amplified from 1 µs to 2 µs the shape of the fin changes from straight towards tilted. When the wire electrode completes its travel along the left side there was no evidence of these bends because at this instant the right side is fully solid and the sufficient amount of substrate material is available to dissipate the process generated heat into the bulk material. As the wire electrode completes its traverse movement along the left side of the fin and travels along the horizontal direction (along the fin thickness), again no bending phenomenon is experienced. But, as the wire starts moving downside the developing fin (see Figure 1b) the substrate material available along the fin substantially reduced because of the development of the fin thickness. At this moment, the heat needs to be dissipated inside the fin thickness (thickness is low in case of 2 µs pulse on-time as compared with 1 µs on-time). It is a well-known fact of EDM that inside the localized spark area a plasma plume is generated having a temperature over 10,000 °C within the radius of the plasma. The plasma plume/channel builds a pressure that produces non-macro forces which are generally neglected during conventional WEDM [34]. Such non-macro forces may have considerable influence if the targeted machining section is of micro-sized (thin section like micro-fins). The pressure of the plasma plume is shared by the wire electrode and the propagating fin. Since, in the literature it is concluded that the workpiece during WEDM is always under very high pressure produced by the plasma channel [35], therefore under the high pressure and non-macro forces a bending action tends to initiate (similar to cantilever beam) and consequently the bends are experienced at the apex of the fins. The electric sparks produced during WEDM cause a thermal deformation, especially when the machined feature is very thin, and the result is geometrical inaccuracies [36]. The results of IFH experienced during experiments 19 through 27 are relatively discouraging since the inter-channels fins are completely curly with very small height. The main reason is the higher level of pulse on-time (3 µs). This is the extension of the previously discussed phenomenon. In this case, the bending action is superimposed by the high discharge energy density caused by the high value of T-on. As the on-time of the discharge pulses is increased the large thermal shocks are produced due to the thermal stresses and as a result, high material removal is observed. Hence, as the discharge energy is increased the inter-channels fin thickness (IFT) is significantly reduced and the material available to digest the discharge heat remains extremely low. The higher amount of heat is dissipated inside the thinner fin and the opposite side of the fin instantly reaches at the same temperature due to its good conductivity (see Table 1). In this way, the top regions of the fin experience a partial melting and the result is reduced IFH. In some cases, the fins are severely damaged as can be witnessed in Figure 7j,k. That is why the spread of data points associated with IFH did not follow the normal distribution and some points, shown in Figure 3c, fall away from the good-fit line.

#### 3.3.3. Analysis of Interchannels Fin Radius (IFR)

**Figure 8:**
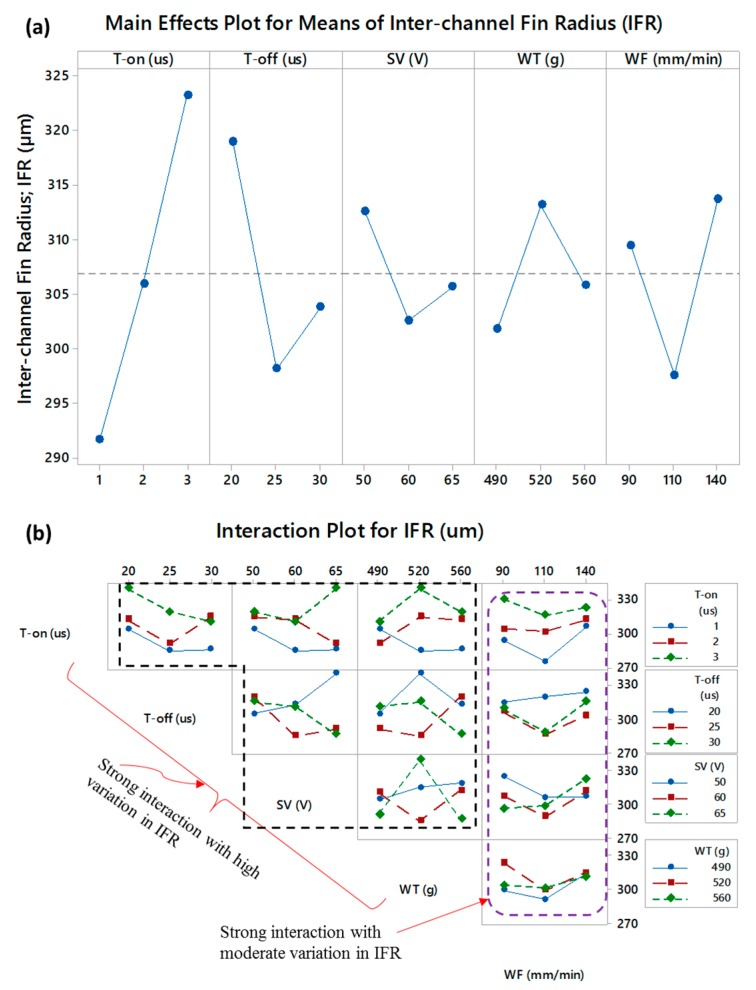
Parametric effects on inter-channel fin radius (IFR): (**a**) main effects plot and (**b**) interaction plot.

The parametric effects on inter-channels fin radius (IFR) are presented in Figure 8. The radii on both sides of a fin are measured and the average radius is reported in this study. The radius associated with the inter-channels fin fluctuates within 257–343 µm. Under the set of parametric conditions employed in this research, the radius associated with the inter-channels fin (IFR) fluctuates within 257–343 µm, i.e., a variation of 90 µm is observed in IFR. In this variation, all the parameters contribute to their individual role as well as their interacting role. With the increase in T-on, the radius of the fabricated fin is increased. As the T-off is increased from 20 µs to 25 µs, the IFR is significantly reduced and the resulting fins have relatively sharp corners at the bottom end. But a further increase in off-time (30 µs) slightly uplifts the IFR. Servo voltage also explains the similar effect. With reference to the wire tension, the radius of the fin is greater when the WT is at 520 g compared with the radius against 490 g wire tension. An increase in the wire tension tends to maintain a uniform inter-electrodes gap and the result is that the radius of the machined curvature is increased. Upon further increase in wire tension, a high probability of wire vibration does exist and the uniformity of the inter-electrode gap is somehow compromised. In this way, the IFR is lower. Similar results are reported in [25] where it is stated that as the wire tension is increased the accuracies in the corner radii also improved. The wire tension with lower values deviates the wire from the machining path and imparts inaccuracy at the corner. The trend line of the wire feed rate and its effect on IFR are opposite to those in the case of wire tension. The middle level of the wire feed rate seems to be appropriate to keep the IFR at the minimum value. The trend lines of each of the five machining variables are in close competition among each other. Therefore, it was necessary to rank the contributing variable. Since among the trend lines in the main effects plots show a considerable effect on the values of IFR, therefore similar results are obtained during the S/N ratio in terms of delta values. Table 8 shows the S/N ratio analysis associated with IFR. Since the designed configurations of the micro-channels and the fins are in such a way that the side-walls are perpendicular to the base surface, therefore, smaller is the better criterion selected for IFR. Based on the delta values, T-on, T-off, and WF are the top three ranked variables, respectively; whereas, the WT and SV acquired 4th and 5th rank with respect to their effect on IFR. The parametric effects on IFR in terms of interactions are shown in Figure 8b. It can be envisioned that all the five machining parameters strongly interact with each other since in each of the subset of Figure 8b the trend lines are non-parallel and intersecting with each other. However, the variation imparted in IFR by the wire feed rate (WF) is found to be moderate as compared to the interaction effects of the remaining four parameters.

#### 3.3.4. Analysis of Micro-Channels Width (MCW)

**Figure 9:**
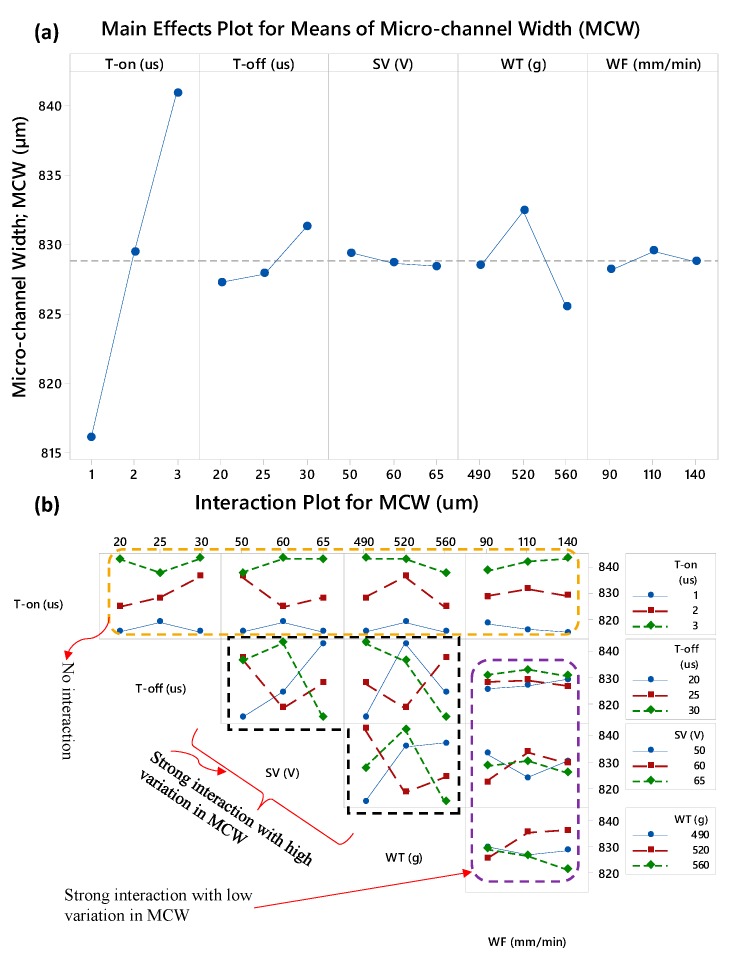
Parametric effects on micro-channel’s width (MCW): (**a**) main effects plot and (**b**) interaction plot.

With reference to the micro-channel geometry, laser beam machining (LBM) produces the channels with taperness along the sidewalls. The width of the channel at the bottom end becomes less than the width at the top due to the presence of sidewalls’ taper angle [37]. In some cases, bottom width even diminishes and V-shaped channels are produces. In this way the overall geometry of the micro-channel is compromised [38]. Thus, another advantage of the WEDM is that the width of the micro-channel remains uniform and less of a compromise on the channels geometry can be experienced. Figure 9 represents the main effects and interaction effects of the WEDM variables on the micro-channel width. It should be noted that in a single experimental run, two micro-channels with one inter-channels fin are produced and the average value of the two micro-channels’ width is considered for the analysis. During the micro-channels fabrication through WEDM the width (under L27 experimentation) of 1000 µm was designed. In comparison with the IFT, the value of MCW is substantially high. Therefore, the wire electrode has considerable freedom to travel along the micro-channel’s width. As a result, the machining phenomenon, distributed over this region (MCW), remains simple and conventional as compared with the machining phenomenon involved in the cases of inter-channels fin thickness and height (IFT and IFH). From the statistical description of the response characteristics (as presented in Table 5), it can also be noticed that the minimum width of the micro-channels is 805 µm and the maximum one is 848 µm. It indicates that under the L27 experimental design the variation in micro-channel width (MCW) is around 40 µm. That is why, from Figure 9a, it can be noticed that the influence of all the machining parameters on MCW seems to be insignificant except for the influence of T-on. The pulses delivered for a longer period of time (T-on) widen the micro-channels and the mean of micro-channel width reaches >840 µm when T-on is selected at the value of 3 µs. The result of S/N ratio analysis associated with MCW (as shown in Table 9) also reveals that the delta score of T-on is exceptionally higher than the delta values of the remaining four parameters. The “larger is better” criterion was set during the S/N ratio analysis of MCW. The ranking of WEDM parameters in the context of the micro-channels width follows the sequence of T-on, WT, T-off, WF, and SV as the 1st, 2nd, 3rd, 4th, and 5th rank respectively. With reference to the mutually-interacting effects of the WEDM parameter, the pulse on-time doesn’t interact with anyone of the remaining four parameters (T-off, SV, WT, and WF) as the trend lines do not intersect with each other. However, strong interactions are observed among the four variables in which the wire feed (WF) gives moderate variations in MCW and T-off, SV, and WT offer relatively high variations in MCW.

### 3.4. Correlation Analysis of Micro-Channels Attriutes

It has been observed that the process parameters considerably affect the micro-channels features including IFT, IFH, IFR, and MCW. Therefore, assessing the relationships between the WEDM parameters and the set response characteristics is felt to be necessarily carried out. Thus, the correlation analysis is performed. Each response attribute is tested against each of the five variables. A confidence level of 95% (α = 0.05) is set for the tests. The type of relationship could be either linear or monotonic which is decided by the *p*-value. A *p*-value ≤ α leads to the conclusion that the correlation’s strength is other than zero (zero indicates no correlation) while the *p*-value greater than the α-level shows that the relationship between the variables is equal to zero. The strength of the correlation is based on the correlation coefficient. The value of the coefficient lies between 0 and 1. The coefficient close to or equals to “1” indicates the presence of a strong relationship whereas the coefficient close to or equals to “0” represents the existence of a poor or very weak relationship. The direction of the relationship (positive and/or negative) is decided by the arithmetic sign of the correlation coefficient. It is worth mentioning that before performing the correlation analysis, each of the four response attributes was plotted (scatter plot) against each of the five process parameters to estimate the data point’s distribution around the regression line. Upon the spread of data around the trend line, if the points were uniformly distributed then “Pearson correlation” was performed otherwise in the case of the monotonic point spread, the “Spearman’s rank-order correlation” is performed. The results of correlation analysis performed on each of the four response measures (IFT, IFH, IFR, and MCW) are presented in Table 10.

The *p*-values associated with each of the four responses against the pulse on-time (T-on) are less than the threshold value. It indicates that all the micro-channel’s features follow a substantial relationship with the pulse on-time. The correlation coefficient of 0.639 indicates that the pulse on-time has a moderately strong relationship with IFT. This relationship is a negative linear. The pulse on-time has a positive linear relationship with the inter-channels fin radius (IFR). The strength of this relationship is found to be moderately strong having a coefficient value of 0.596. On the other end, the relationship of T-on with the inter-channels fin height (IFH) is observed to be highly strong with a coefficient of 0.866. However, the direction of this relation is negative. Finally, the micro-channels width (MCW) acquire a strong, positive and linear relationship with T-on.

In a similar pattern, the strength and the direction of the relationships of each response measure with each process parameter are presented in Table 10. As a whole, with the remaining four process parameters (T-off, SV, WT, and WF) none of the response attributes has a strong relationship. However, some weak relationships are obtained, for example, T-off on IFR, and WT on IFH. In many cases, a zero relationship is experienced, for example, the SV has zero relationships with IFT, IFH, and MCW, and the wire feed rate has zero relationships with IFT, IFH, and MCW.

## 4. Conclusions

Wire electric discharge machining of copper has been done to produce the micro-channels. Initially, the minimum size of the micro-channel, which can be easily produced through WEDM, has been determined based on the inter-channels fin thickness. After performing 29 trial experiments, the mature experimentation under Taguchi L27 was performed. The parametric effects of five variables (pulse on-time; T-on, pulse off-time; T-off, servo voltage; SV, wire tension; WT, and wire feed rate; WF) over the four response characteristics (inter-channels fin thickness; IFT, inter-channels fin height; IFH, inter-channels fin radius; IFR, and micro-channel width; MCW) have been evaluated. The main effects and interaction effects are discussed along with the S/N analysis in order to prioritize the effect of EDM variables over the micro-channel’s attributes. Analysis of correlation is also carried out to estimate the relationships between the EDM variables and the response measures. After machining, analysis, results, and discussion, the following conclusions have been inferred:

i. Among the other attributes of the micro-channels, the inter-channels fin thickness (IFT) and the inter-channels fin height (IFH) are more sensitive to the WEDM parameters. The width of the micro-channel (MCW) and the inter-channels fin radius (IFR) can be controlled easily because of having ample space (1000 µm) for the wire to travel along the width of the channels.
ii. The shape of the fin remains intact at the bottom end, whereas the top end of the fin gets damaged due to the pressure of plasma plume and the excessive heat dissipation over the less available material at the fin tip. Therefore, four configurations of inter-channels fins are observed.
  (a) No formation of the inter-channels fin or small spikes (fin broken at the top end);
  (b) Straight fins with moderate deflection (left-sided bend) at the top end;
  (c) Straight fins with aggressive deflection (left-sided curly bend) at the top end;
  (d) Straight fins with no/slight deflection at the top end.
iii. As per the statistical analysis and S/N ratio analysis the pulse on-time (T-on), among the set of five WEDM process parameters, is always found to be the most significant and contributing factor for each of the four attributes of the micro-channels (IFT, IFH, IFR, and MCW). WT and T-off are also observed to have a noticeable effect on most of the micro-channel’s features. SV and WF revealed minimal effects.
iv. The lower level of the pulse on-time (1 µs), the middle level of the pulse off-time (25 µs), and a higher level of the wire tension (560 g) can produce the micro-channels having competitive machining results (straight fins with no distortion) in terms of IFT, IFH, and IFR. However, if the prime concern is to produce the channels with greater width then the high pulse on-time is the most appropriate choice.
v. To get the larger surface area (maximum number of micro-channels per unit area), the inter-channels fin thickness (IFT), fin radius (IFR), and micro-channel width (MCW) should have minimum dimensions, whereas the inter-channels fin height (IFH) should be at its maximum value with respect to the planned dimensions. The micro-channels in which the fins are straight with no deflection at the top end, the minimum values of IFT, IFR and MCW are observed to be 81 µm, 257 µm, and 805 µm, respectively. The maximum value of the inter-channels fin height (IFH), among the results of the straight fin with no deflection, is found to be 939 µm. The multi-objective optimization is expected to be performed in future work which can lead to having more control over the features of micro-channels.

## Figures and Tables

**Table 1:** Thermo-physical properties of copper.

Property	Unit	Value
Density	g/cm^3^	8.96
Hardness	HV	110
Electrical resistivity	Ωm	16.78
Thermal conductivity	W/mK	401
Melting point	°C	1085

**Table 2:** Wire electrical discharge machining (WEDM) parameters utilized during micro-channels screening.

No	Variable Name	Unit	Levels		
			1	2	3
1	Pulse On-time	µs	1	2	3
2	Pulse Off-time	µs	20	25	30
3	Spark Voltage	V	55	60	65
4	Wire Tension	g	490	520	560
5	Wire feed rate	mm/min	90	110	140

**Table 3:** Screening of micro-channel sizes (based on inter-channel fin thickness) and machining parameters.

Runs	Exp. #	Wire Electrical Discharge Machining (WEDM) Parameters	Designed Inter-Channels Fin Thickness; IFT (µm)	Actual Inter-Channels Fin Thickness; IFT (µm)	Remarks
	Trials Set 1: Inter-channel wall thickness ranging from 1000 to 250 µm (an increment of 250 µm)				
**1**	1-1	T-on = 2 µs, T-off = 25 µs, SV = 60 V, WT = 520 g, WF = 110 mm/min	1000	1354	Existence of inter-channel fin with noticeable thickness
**2**	1-2		750	816	
**3**	1-3		500	358	
**4**	1-4		250	-	Inter-channel wall gets broken
	Trials Set 2: Inter-channel wall thickness ranging from 500 to 300 µm (an increment of 50 µm)				
**5**	2-1	T-on = 2 µs, T-off = 25 µs, SV = 60 V, WT = 520 g, WF = 110 mm/min	500	358	Existence of inter-channel fin with noticeable thickness
**6**	2-2		450	239	
**7**	2-3		400	179	
**8**	2-4		350	-	No existence of inter-channel fin
**9**	2-5		300	-	
	Trials Set 3: Inter-channel wall thickness ranging from 400 to 360 µm (an increment of 10 µm)				
**10**	3-1	T-on = 2 µs, T-off = 25 µs, SV = 60 V, WT = 520 g, WF = 110 mm/min	400	179	
**11**	3-2		390	-	No existence of inter-channel fin
**12**	3-3		380	-	
**13**	3-4		370	-	
**14**	3-5		360	-	
	Trials Set 4: Inter-channel wall with constant thickness of 400 µm (confirmatory runs)				
**15**	4-1	T-on = 2 µs, T-off = 25 µs, SV = 60 V, WT = 520 g, WF = 110 mm/min	400	Measurements were not performed. Only CMM-based observations were captured	Inter-channel fin having a bend at the tip
**16**	4-2		400		
**17**	4-3		400		
**18**	4-4		400		
**19**	4-5		400		
	Trials Set 5: Inter-channel wall with constant thickness of 400 µm (confirmatory runs)				
**20**	5-1	T-on = 3 µs, T-off = 30 µs, SV = 65 V, WT = 560 g, WF = 140 mm/min	400	-	No existence of inter-channel fin
**21**	5-2		400	-	
**22**	5-3		400	-	
**23**	5-4		400	-	
**24**	5-5		400	-	
	Trials Set 6: Inter-channel wall with constant thickness of 400 µm (confirmatory runs)				
**25**	6-1	T-on = 1 µs, T-off = 20 µs, SV = 55 V, WT = 490 g, WF = 90 mm/min	400	Measurements were not performed. Only CMM-based observations were captured	Straight inter-channel fins were obtained.
**26**	6-2		400		
**27**	6-3		400		
**28**	6-4		400		
**29**	6-5		400		

**Table 4:** Experimental results in terms of micro-channels attributes under Taguchi L27.

Run	WEDM Parameters					Responses (Micro-Channels’ Attributes)			
	T-on (µs)	T-off (µs)	SV (V)	WT (g)	WF (mm/min)	IFT (µm)	IFR (µm)	IFH (µm)	MCW (µm)
1	1	20	50	490	90	95	304.5	936	822.5
2	1	20	50	490	110	115	299	863	805
3	1	20	50	490	140	90	309	921	817
4	1	25	60	520	90	107	303	902	809.5
5	1	25	60	520	110	98	257	939	825.5
6	1	25	60	520	140	81	294.5	915	821
7	1	30	65	560	90	94	274.5	923	822
8	1	30	65	560	110	84	270.5	922	817
9	1	30	65	560	140	116	313.5	929	806
10	2	20	60	560	90	101	301.5	923	821
11	2	20	60	560	110	62	318	949	830
12	2	20	60	560	140	79	318	897	822.5
13	2	25	65	490	90	72	277.5	819	830.5
14	2	25	65	490	110	87	284	851	829
15	2	25	65	490	140	96	311	890	824
16	2	30	50	520	90	70	334	826	833.5
17	2	30	50	520	110	70	302	886	835
18	2	30	50	520	140	65	308	825	840
19	3	20	65	520	90	82	336	491	833.5
20	3	20	65	520	110	72	340.5	387	846
21	3	20	65	520	140	59	343.5	491	848.5
22	3	25	50	560	90	74	337	673	844.5
23	3	25	50	560	110	82	316	536	832.5
24	3	25	50	560	140	90	303.5	550	835
25	3	30	60	490	90	71	317.5	611	837
26	3	30	60	490	110	68	291.5	528	846.5
27	3	30	60	490	140	60	322.5	383	845.5

**Table 5:** Descriptive statistics of responses.

Responses	N	Min.	Max.	Mean	StDev
IFT (µm)	27	59	116	82.96	16.06
IFR (µm)	27	257	343.5	306.9	21.93
IFH (µm)	27	383	949	769.1	192.20
MCW (µm)	27	805	848.5	828.9	12.20

**Table 6:** Signal-to-noise (S/N) ratio analysis of IFT (Larger is better).

Level	T-on (µs)	T-off (µs)	SV (V)	WT (g)	WF (mm/min)
1	39.74	38.29	38.30	38.30	38.49
2	37.73	38.77	37.97	37.72	38.13
3	37.20	37.62	38.41	38.65	38.05
Delta	2.54	1.15	0.44	0.93	0.44
Rank	1	2	5	3	4

**Table 7:** S/N ratio analysis of IFH (Larger is better).

Level	T-On (µs)	T-Off (µs)	SV (V)	WT (g)	WF (mm/min)
1	59.24	57.19	57.67	57.24	57.76
2	58.82	57.73	57.50	56.98	57.26
3	54.13	57.27	57.02	57.98	57.17
Delta	5.11	0.54	0.64	1.00	0.59
Rank	1	5	3	2	4

**Table 8:** S/N ratio analysis of IFR (smaller is better).

Level	T-on (µs)	T-off (µs)	SV (V)	WT (g)	WF (mm/min)
1	−49.28	−50.06	−49.89	−49.59	−49.79
2	−49.70	−49.47	−49.60	−49.88	−49.44
3	−50.18	−49.63	−49.67	−49.69	−49.92
Delta	0.89	0.60	0.29	0.30	0.48
Rank	1	2	5	4	3

**Table 9:** S/N ratio analysis of MCW (Larger is better).

Level	T-On (µs)	T-Off (µs)	SV (V)	WT (g)	WF (mm/min)
1	58.24	58.35	58.37	58.37	58.36
2	58.38	58.36	58.37	58.41	58.38
3	58.50	58.40	58.36	58.33	58.37
Delta	0.26	0.04	0.01	0.07	0.01
Rank	1	3	5	2	4

**Table 10:** Correlation analysis of micro-channels’ attributes with electric discharge machining parameters.

Response	*p*-Value	Significance	Coefficient	Relationship	
				Strength	Direction
**Correlation of micro-channels’ features with pulse on-time (T-on)**					
**IFT**	0.000	Significant	−0.639	Moderately strong	-ve
**IFR**	0.001	-	0.596	Moderately strong	+ve
**IFH**	0.000	-	−0.866	Strong	-ve
**MCW**	0.000	-	0.847	Strong	+ve
**Correlation of micro-channels’ features with pulse off-time (T-off)**					
**IFT**	0.414	Insignificant	−0.164	Very weak	-ve
**IFR**	0.147	-	−0.287	Weak	-ve
**IFH**	0.976	-	−0.006	Zero	NA
**MCW**	0.492	-	0.138	Very weak	+ve
**Correlation of micro-channels’ features with servo voltage (SV)**					
**IFT**	0.950	-	0.013	Zero	NA
**IFR**	0.439	-	−0.155	Very weak	-ve
**IFH**	0.751	-	−0.064	Zero	NA
**MCW**	0.868	-	−0.033	-	-
**Correlation of micro-channels’ features with wire tension (WT)**					
**IFT**	0.628	-	0.098	Zero	NA
**IFR**	0.771	-	0.059	-	-
**IFH**	0.522	-	0.129	Very weak	+ve
**MCW**	0.559	-	−0.118	-	-ve
**Correlation of micro-channels’ features with wire feed rate (WF)**					
**IFT**	0.689	-	−0.081	Zero	NA
**IFR**	0.569	-	0.155	Very weak	+ve
**IFH**	0.731	-	0.069	Zero	NA
**MCW**	0.938	-	0.016	-	-
